# Interventions to prevent hemodynamic instability during renal replacement therapy in critically ill patients: a systematic review

**DOI:** 10.1186/s13054-018-1965-5

**Published:** 2018-02-22

**Authors:** Adrianna Douvris, Gurpreet Malhi, Swapnil Hiremath, Lauralyn McIntyre, Samuel A. Silver, Sean M. Bagshaw, Ron Wald, Claudio Ronco, Lindsey Sikora, Catherine Weber, Edward G. Clark

**Affiliations:** 1 Department of Medicine, University of Ottawa, Ottawa, ON Canada; 2 Division of Nephrology, Department of Medicine and Kidney Research Centre, Ottawa Hospital Research Institute, University of Ottawa, Ottawa, ON Canada; 3 Division of Critical Care, Department of Medicine, The Ottawa Hospital, Ottawa, ON Canada; 4 Centre for Transfusion Research, Clinical Epidemiology Program, Ottawa Hospital Research Institute, Ottawa, ON Canada; 5 Division of Nephrology, Queen’s University, Kingston, ON Canada; 6 Department of Critical Care Medicine, Faculty of Medicine and Dentistry, University of Alberta, Edmonton, AB Canada; 7 Division of Nephrology, St. Michael’s Hospital, Toronto, ON Canada; 8 International Renal Research Institute and Department of Nephrology, St. Bortolo Hospital, Vicenza, Italy; 9 Health Sciences Library, University of Ottawa, Ottawa, ON Canada; 10 Division of Nephrology, McGill University, Montreal, Quebec Canada; 11 The Ottawa Hospital – Riverside Campus, 1967 Riverside Drive, Ottawa, ON K1H 7W9 Canada

**Keywords:** Acute kidney injury, Renal replacement therapy, Intradialytic hypotension, Dialysis, Hemodynamic instability

## Abstract

**Background:**

Hemodynamic instability related to renal replacement therapy (HIRRT) may increase the risk of death and limit renal recovery. Studies in end-stage renal disease populations on maintenance hemodialysis suggest that some renal replacement therapy (RRT)-related interventions (e.g., cool dialysate) may reduce the occurrence of HIRRT, but less is known about interventions to prevent HIRRT in critically ill patients receiving RRT for acute kidney injury (AKI). We sought to evaluate the effectiveness of RRT-related interventions for reducing HIRRT in such patients across RRT modalities.

**Methods:**

A systematic review of publications was undertaken using MEDLINE, MEDLINE in Process, EMBASE, and Cochrane’s Central Registry for Randomized Controlled Trials (RCTs). Studies that assessed any intervention’s effect on HIRRT (the primary outcome) in critically ill patients with AKI were included. HIRRT was variably defined according to each study’s definition. Two reviewers independently screened abstracts, identified articles for inclusion, extracted data, and evaluated study quality using validated assessment tools.

**Results:**

Five RCTs and four observational studies were included (*n* = 9; 623 patients in total). Studies were small, and the quality was mostly low. Interventions included dialysate sodium modeling (*n* = 3), ultrafiltration profiling (*n* = 2), blood volume (*n* = 2) and temperature control (*n* = 3), duration of RRT (*n* = 1), and slow blood flow rate at initiation (*n* = 1). Some studies applied more than one strategy simultaneously (*n* = 5). Interventions shown to reduce HIRRT from three studies (two RCTs and one observational study) included higher dialysate sodium concentration, lower dialysate temperature, variable ultrafiltration rates, or a combination of strategies. Interventions not found to have an effect included blood volume and temperature control, extended duration of intermittent RRT, and slower blood flow rates during continuous RRT initiation. How HIRRT was defined and its frequency of occurrence varied widely across studies, including those involving the same RRT modality. Pooled analysis was not possible due to study heterogeneity.

**Conclusions:**

Small clinical studies suggest that higher dialysate sodium, lower temperature, individualized ultrafiltration rates, or a combination of these strategies may reduce the risk of HIRRT. Overall, for all RRT modalities, there is a paucity of high-quality data regarding interventions to reduce the occurrence of HIRRT in critically ill patients.

**Electronic supplementary material:**

The online version of this article (10.1186/s13054-018-1965-5) contains supplementary material, which is available to authorized users.

## Background

Hemodynamic instability related to renal replacement therapy (HIRRT) is a frequent occurrence in critically ill patients with acute kidney injury (AKI) [1]. HIRRT complicates an estimated 30–70% of intermittent hemodialysis (IHD) treatments for AKI in the intensive care unit (ICU) [2–4]. HIRRT is also a frequent complication of other renal replacement therapy (RRT) modalities, specifically sustained low-efficiency dialysis (SLED) and continuous renal replacement therapy (CRRT) [5]. CRRT is presumed to have the least impact on the hemodynamic stability of critically ill patients [6]; nonetheless, RRT-related hypotension has still been reported to occur in 19 to 43% of patients treated with CRRT [7, 8].

There is evidence suggesting that HIRRT negatively impacts outcomes for patients with RRT-requiring AKI; more frequent HIRRT is associated with increased mortality [9] and may limit renal recovery after AKI [10]. Hypotensive episodes during RRT lead to decreased renal perfusion and may compromise renal recovery on that basis [10]. Accordingly, interventions to limit HIRRT across RRT modalities might ultimately improve the persistently dismal outcomes of critically ill patients with AKI treated with RRT [11–13]. As such, we sought to assess the efficacy and harms of RRT-related interventions for preventing or mitigating HIRRT in critically ill patients with AKI.

## Methods

This systematic review was conducted according to a previously published protocol [14] and was registered with PROSPERO (PROSPERO 2016:CRD42016037754). A summary of the study methods follows.

### Study population

We conducted a systematic review of published studies, including interventional and observational studies of critically ill adults in a medical or surgical ICU with AKI treated with RRT. Studies that involved IHD, SLED, or CRRT were included. Studies were excluded if they involved the end-stage renal disease (ESRD) population or peritoneal dialysis. Case reports, animal experiments, non-English language reports, and studies directly comparing RRT modalities were excluded.

### Intervention

All included studies had a dialysis-related intervention or modifiable factor related to the application of RRT to prevent or mitigate HIRRT [14]. In a minor deviation from the previously published protocol for this systematic review [14], studies comparing dialysate buffers and filter membranes were excluded given that the use of bicarbonate-based buffers and biocompatible membranes is now standard in contemporary practice. Studies that did not include any prescribed intervention/modifiable factor were also excluded.

### Comparator

Our comparators were the groups of patients in these studies that did not receive the intervention. Observational studies without a comparator group were excluded.

### Outcomes

The primary outcome was HIRRT according to the definitions provided in the individual studies. Secondary outcomes included death, ICU and hospital length of stay, renal recovery, need for interventions (vasopressor dose change, need for fluid bolus, reduced ultrafiltration goal, or cessation of ultrafiltration) to treat HIRRT, cardiovascular events, system clotting, and bleeding. We also assessed for intervention-specific harms or side effects.

### Study identification

A comprehensive search strategy was developed with, and implemented by, a health information specialist (LS). Our published protocol describes the search strategy in detail [14]. An initial search of MEDLINE, PubMed, and PROSPERO yielded no prior or ongoing systematic reviews on this topic. Our search accessed the following databases: MEDLINE in Process and MEDLINE (via OVID), Embase (via OVID), and CENTRAL (via OVID). The cutoff date was 26 April 2017. To supplement our search, we also searched PubMed, reference lists, conference abstracts, and clinical trial registries. The PubMed search captured one additional publication that was missed by the initial search strategy and so the search strategy was expanded and re-run but did not yield any further articles for inclusion, including the initially missed article (which was still missed by the expanded strategy due to ‘acute kidney injury’ or ‘acute renal failure’ not having been used as a keyword or in the title of that particular publication).

### Study selection and quality assessment

Two reviewers (AD, EGC) independently screened the study reference database for potentially eligible studies. Studies deemed potentially eligible underwent full text review. Any disagreements were resolved by consensus or discussion with a third investigator (SH). We used the Newcastle-Ottawa Quality Assessment Scale (NOS) [15] and the Cochrane Collaboration’s Tool for Assessing Risk of Bias in Randomised Trials [16] for the quality assessment of observational studies and randomized controlled trials (RCTs), respectively. For RCT quality assessment, the risk of bias was reported as low, unclear, or high risk as described by Higgins et al. [16].

### Data extraction and synthesis

Two reviewers (AD, EGC) independently extracted data from all included studies. We created data extraction forms to record the following information from each study: author, year, type of study, population characteristics, intervention and comparator group, and primary and secondary outcomes. Given the small number of studies and large heterogeneity between studies, as was expected [14], we were unable to perform a meta-analysis and have presented our data as a narrative synthesis.

## Results

**Fig. 1:**
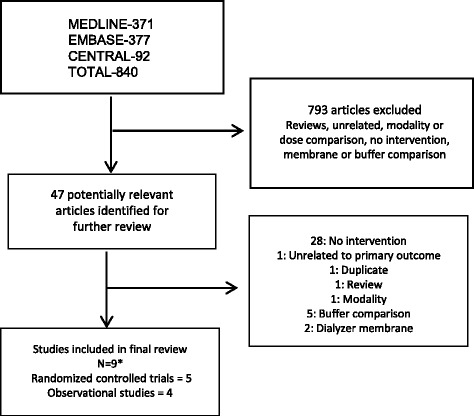
Flow diagram of included studies and exclusions. Initial search of MEDLINE/PubMed and Prospero yielded no prior or ongoing systematic reviews on this topic. A health information specialist constructed and implemented the comprehensive search strategy. *Not included in the diagram: one RCT that meets inclusion criteria was not identified using this search strategy but rather, using a PubMed search, likely because the term ‘acute kidney injury’ or ‘acute renal failure’ was not in the title or listed as a keyword, and our search strategy was designed to capture studies of acute kidney injury and renal replacement therapy. Given the missed study, the search strategy was expanded, and identified 181 additional articles. Again, the same study was missed for the reason above. Five additional studies from the second search underwent full text review but were ultimately excluded because they included dose comparisons, dialysate buffer, and dialyzer membrane comparisons

The search process and results are depicted in Fig. 1.

A total of 840 citations were identified, of which 793 were excluded based on title and abstract. Forty-seven studies underwent full text review. Of these, 28 were excluded because there was no comparator group, five were dialysate buffer comparisons, two involved dialyzer membranes, one was unrelated to the study topic, one was a modality comparison, and one was a systematic review of bicarbonate versus lactate-buffered solutions for AKI treated with RRT [17].

In total, nine studies, consisting of five RCTs and four observational studies, met inclusion criteria and are summarized in Tables 1 and 2. Study sizes ranged from as small as 10 patients to as large as 191 patients, for a total of 623 patients. Interventions included dialysate sodium modeling (*n* = 3), ultrafiltration profiling (*n* = 2), blood volume (*n* = 2) and temperature control (*n* = 3), duration of RRT (*n* = 1), and slow blood flow rate at initiation (n = 1). Some studies applied more than one strategy simultaneously (*n* = 5).

**Table 1:** Summary of study designs, outcomes, and definitions of HIRRT

Study	Setting and country	Intervention	Study design	Sample size	Mean age	Male (%)	Primary outcome(s)	HIRRT definition
*Intermittent hemodialysis*								
Lynch (2016) [21]	USA Single center Medical/surgical ICU	Dialysate Na^+^ modeling	Retrospective cohort	*n* = 191 RRT = 892 (242/892 Na modeling)	62 ± 17	60.7	In-hospital death *or* dialysis dependence at dischargeHIRRT	SBP < 80 mmHg, *or* 50 mmHg drop from pre-HD BP, *and/or* start of vasopressor during HD
du Cheyron (2013) [20]	France Single center Medical ICU	Blood volume and temperature control	RCT	*n* = 74 RRT = 574	65 ± 10	68	HIRRTArrhythmias RRT-related complication	SBP < 90 mmHg justifying intervention
du Cheyron (2010) [19]	France Single center Medical ICU	Blood volume and temperature control	Prospective cohort	*n* = 62 RRT = 572	60 (57–70)	48.4	HIRRTInterventionsArrhythmias	SBP < 90 mmHg or fall > 40 mmHg
Schortgen (2000) [22]	France Single center Medical ICU	“Guidelines” for IDH in AKI	Retrospective cohort	*n* = 121 RRT = 537	57–60 ± 15	25.6	HIRRT, intervention, length of stay, mortality	SBP drop > 10% from baseline or infusion need
Paganini (1996) [26]	USA Single center ICU^*^	Variable dialysate Na^+^ and UF modeling	RCT with crossover design	*n* = 10 RRT = 60	64.2 ± 13.7	80	HemodynamicsVolume removal, blood volume change	Interventions: volume ± vasopressors
*Sustained low-efficiency dialysis*								
Albino (2014) [24]	Brazil Single center ICU*^¶^	Duration of dialysis: 6 vs 10 h	RCT	*n* = 75 RRT = 195	61.8 ± 15.1	70.6	HIRRT, renal recovery, mortality	SBP < 90 mmHg MAP < 60 mmHg
Lima (2012) [23]	Brazil Single center Medical ICU	Lower temperature, dialysate Na^+^ and UF profiling	RCT	n = 39 RRT = 62	58 ± 16	67.7	HIRRT, length of stay, mortality	SBP < 90 mmHg MAP < 60 mmHg Interventions
*Continuous renal replacement therapy*								
Robert (2012) [25]	France Single center Medical/surgical ICU	Temperature	RCT with crossover design	*n* = 30 time = 12 h	66.5 ± 10.3	70	Hemodynamic tolerance	Fall in MAP > 20% or intervention
Eastwood (2012) [18]	Australia Single center ICU*	CRRT pump speed	Prospective cohort	*n* = 21 RRT = 41 starts	58+/−19.9	48	Hemodynamic parameters	Vasopressors, fluid bolus at 10, 30 min Hypotension not defined

*Type of ICU (medical, surgical, or both) not specified

^¶^All included patients had acute kidney injury (AKI) associated with sepsis, and were on a norepinephrine infusion (0.3–0.7 μg/kg/min)

*CRRT* continuous renal replacement therapy, *HD* hemodialysis, *HIRT* hemodynamic instability during renal replacement therapy, *MAP* mean arterial pressure, *Na*^+^ sodium, *RCT* randomized controlled trial, *RRT* renal replacement therapy, *SBP* systolic blood pressure, *SLED* sustained low-efficiency dialysis, *UF* ultrafiltration

**Table 2:** Comparison of renal replacement therapy prescriptions, achieved ultrafiltration goals, and duration of treatment

Study	Q_B_ (mL/min)	Temperature (°C)*	UF rate (mL/h)	UF goal (L)	UF achieved (L)	Dialysate Na^+^ (mmol/L)	Dialysate Ca^+^ (mmol/L)	Time (h)*
*Intermittent hemodialysis*								
Lynch (2016) [21]	Case: 310 Control: 292	“Cooled dialysate” (%) Case: 12 Control: 2.3	Not specified	Median: 2.25	Mean 2 L No difference; 38% of sessions did not reach goal	Modeling: not specified Fixed: 140	1.25	Case: 3.37 Control: 3
du Cheyron (2013) [20]	200–250	1 °C below body temperature	BVM: 500 BVM + BTM: 522 Control: 500	Not specified	BVM: 3.0 BVM + BTM: 3.0 Control: 3.0	145	1.75	> 4
du Cheyron (2010) [19]	200–250	36.0	Case: 548 ± 92 Control: 415 ± 112	Not specified	Case: 3.0 ± 0.64 Control: 2.1 ± 0.62	145	1.75	> 4
Schortgen (2000) [22]	150–200	“Guidelines”: ≤ 37.0 Control: ≥ 37.0	Not specified.“Sequential UF” in 15% of cases	Not specified	“Guidelines”: −11 ± 515 mL Control: +135 ± 434 mL	“Guidelines”: > 145 in 67% Control: < 145	1.75	“Guidelines”: 5.0 ± 1.5 Control: 4.2 ± 1.0
Paganini (1996) [26]	300	Unknown	Variable (Case) vs Fixed (Control)	Not specified	Case: 2.0 ± 1.2 L Control: 1.56 ± 1.3 L	Case: 160 to 140 Fixed: 140	Unknown	> 4
*Sustained low-efficiency dialysis*								
Albino (2014) [24]	200	35.5	Case: 221–237 Control: 288–357	Case: 2.52–2.76Control: 1.95–2.26	Case: 2.21–2.37 Control: 1.73–2.14	Range 142–148	Unknown	Case: 10 Control: 6
Lima (2012) [23]	150–200	Case: 35.5 Control: 37.0	Case: Variable Control: Fixed	Not specified	Case: 2.23 ± 1.2 Control: 1.59 ± 1.0	Case: 150 to 138 Control: 138	1.75	> 6
*Continuous renal replacement therapy*								
Robert (2012) [25]	150–200	Heating device at 36.0 or 38.0 then crossover at 6 h	35 mL/kg/h	Not specified	Not specified	Not specified	Not specified	Not specified (Time period for outcomes assessment: first 12 h after initiation)
Eastwood (2012) [18]	Routine: increase of 50 mL/min over 1–4 min until 200 mL/min Slow: increase of 20–50 mL/min over 3–10 min until 200 mL/min	Not specified	Not specified	Not specified	Not specified	Not specified	Not specified	Not specified (Time period for outcomes assessment: first 30 min after initiation)

*Unless otherwise specified

The term ‘case’ is used to refer to the group that received an intervention to limit hemodynamic instability related to renal replacement therapy (HIRRT), irrespective of study design

*BTM* blood temperature online monitoring, *BVM* blood volume online monitoring, *Ca* calcium, *Na* sodium, *Q*_*B*_ blood flow rate, *UF* ultrafiltration

Table 3 reports on the overall incidence of HIRRT across studies. Notably, no studies specifically assessed potential adverse effects (or side effects) of interventions to prevent HIRRT.

**Table 3:** Study features and overall incidence of HIRRT

Study	HIRRT definition	Intervention	Severity of illness scores	Pre-dialysis BP (mmHg)*	HIRRT^¶^
*Intermittent hemodialysis*					
Lynch (2016) [21]	SBP < 80 mmHg, *or* 50 mmHg drop from pre-HD BP, *and/or* start of vasopressor during HD	Dialysate sodium modeling	*SOFA:* Case: 13.0 ± 2.0 Control: 13.0 ± 3.0	Case: 119.0 ± 16.0 Control: 129.0 ± 21.0	Case: 36/242 = 14.9% Control: 59/650 = 9.1% Overall: 95/892 = 10.6%
du Cheyron (2013) [20]	SBP < 90 mmHg justifying Intervention	BVM and BTM	*SOFA:* BVM: 7 (5–9) BVM + BTM: 8 (4–11) Control: 8 (5–10) Overall: 10 (8–12)	Not reported for start of sessions but “did not differ among treatment modalities at any time”	BVM: 33/190 = 17.4% BVM + BTM: 30/194 = 15.5% Control: 32/188 = 17.0% Overall: 95/572 = 16.6%
du Cheyron (2010) [19]	SBP < 90 mmHg or fall> 40 mmHg	Blood volume and Temp control	*SOFA:* Case: 8.5 (6–16)Control: 8.0 (5–14)	Not reported	Case: 41/189 = 21.7% Control: 110/383 = 28.7% Overall: 151/572 = 26.4
Schortgen (2000) [22]	SBP drop > 10% from baseline *or* volume *or* vasopressors	“Guidelines” for HIRRT in AKI	*SAPS II:*“Guidelines”: 59.0 ± 24.0 Control: 50.0 ± 17.0	“Guidelines”: 121.0 ± 23.0 Control: 125.0 ± 24.0	“Guidelines”: 176/289 = 60.9% Control: 176/248 = 71.0% Overall: 352/537 = 65.5%
Paganini (1996) [26]	Case: volume ± vasopressors	Variable dialysate sodium and UF modeling	*APACHE II:* Overall: 28.7 ± 4.7	MAP: Case: 82.8± 16.9 Control: 86.2± 18.9	Case: 16.0%^§^ Control: 45.4%^§^
*Slow low-efficiency dialysis*					
Albino (2014) [24]	SBP < 90 mmHg MAP < 60 mmHg	Duration of dialysis 6 vs 10 h	*SOFA:* 6 h: 13.1 ± 2.4 10 h: 14.2 ± 3.0 Overall: 13.6 ± 2.7	Not reported	6 h: 63/100 = 63.0% 10 h: 53/95 = 55.8% Overall: 116/195 = 59.5%
Lima (2012) [23]	SBP < 90 mmHg MAP < 60 mmHg Interventions	Lower temperature, dialysate sodium and UF profiling	*SOFA:* Case: 12.0 ± 3.9Control: 11.0 ± 4.4	Case: 132.0± 25.0 Control: 124.0± 24.0	Case: 8/34 = 23.5% Control: 16/28 = 57.1% Overall: 24/62 = 38.7%
*Continuous renal replacement therapy*					
Robert (2012) [25]	Therapeutic intervention for hypotension	Temperature setting:^♯^ A: 38 °C then 36 °C B: 36 °C then 38 °C	*SOFA:* A:12.8 ± 3.8 B: 8.0 ± 3.8 Overall: 10.6 ± 4.6	A: 118.0 ± 26.0 B: 113.0 ± 26.0 Overall: 117 ± 30	Patients requiring intervention for HIRRT:^♯^ Period 1: A: 8/16 = 50.0% B: 5/14 = 35.7% Period 2: A: 3/11 = 27.3% B:4/11 = 63.6%
Eastwood (2012) [18]	Vasopressor use and/or fluid bolus at 10 and 30 min	CRRT pump speed	*APACHE II:* Case: 23.1 ± 4.5Control: 25.9 ± 6.6 Overall: 24.5± 5.8	MAP: Case: 82.5 ± 15.0 Control: 82.4 ± 15.1 Overall: 82.4 ± 15.0	No HIRRT reported

*Systolic blood pressure, unless otherwise specified

^¶^Incidence per session (rather than per patient), unless otherwise specified

^§^Exact number of HIRRT events/intermittent hemodialysis sessions per group was not reported

^♯^Cross-over after 6 h (period 1 is first 6 h; period 2 is second 6 h)

The term ‘case’ is used to refer to a group that received an intervention to limit HIRRT, irrespective of study design

*AKI* acute kidney injury, *APACHE* Acute Physiology and Chronic Health Evaluation, *BP* blood pressure, *BTM* blood temperature online monitoring, *BVM* blood volume online monitoring, *CRRT* continuous renal replacement therapy, *HD* hemodialysis; *HIRRT* hemodynamic instability during renal replacement therapy, *MAP* mean arterial pressure, *SAPS* Simplified Acute Physiology Score, *SBP* systolic blood pressure, *SOFA* Sequential Organ Failure Assessment, *UF* ultrafiltration

Quality assessment, using the NOS for observational studies, is reported in Table 4 and the Cochrane Collaboration risk of bias for RCTs is reported in Table 5. For the observational studies, three received at least 7/9 stars. For the RCTs, none were considered to have low risk of bias and some types of bias could not be determined based on the information provided.

**Table 4:** Newcastle Ottawa Scale (NOS) for quality assessment of nonrandomized studies

Study	Study design	Selection				Comparability		Outcome			Total points
		S1	S2	S3	S4	C1	C2	O1	O2	O3	
Lynch (2016) [21]	Retrospective cohort	1	1	1	1	1	1	1	1	1	9
du Cheyron (2010) [19]	Prospective cohort	1	0	1	1	1	1	1	1	1	8
Schortgen (2000) [22]	Retrospective cohort	1	0	1	0	0	1	1	1	1	6
Eastwood (2012) [18]	Prospective cohort	1	1	1	1	0	1	0*	1	1	7

*Unclear if blinded assessment

For quality assessment, > 7 points is considered ‘good quality’

**Table 5:** Quality assessment of randomized controlled trials using Cochrane Collaboration’s Tool for Assessing Risk of Bias in Randomized Trials

Study	Selection		Performance	Detection	Attrition	Reporting
	R	A				
Albino (2014) [24]	?	–	+	?	–	–
du Cheyron (2013) [20]	–	–	+	?	+	–
Lima (2012) [23]	–	?	+	?	–	–
Robert (2012) [25]	–	?	+	?	+	–
Paganini (1996) [26]	?	?	+	?	–	?

+ High risk of bias, − low risk of bias, ? unknown risk of bias (moderate is not an option); *A* allocation concealment, *R* random sequence generation

Study designs, outcomes, and definitions of HIRRT used by the included studies are summarized in Table 1. There was wide variability in the definition of HIRRT used in studies both within and across the RRT modalities involved. This was particularly evident for the studies involving IHD, where each used a different definition. There was a consistent definition used by the two studies employing SLED, defining HIRRT as a systolic blood pressure (SBP) < 90 mmHg or mean arterial pressure (MAP) < 60 mmHg. HIRRT definitions were different for the two studies employing CRRT; Eastwood et al. [18] used a definition that included only assessing the need for vasopressors or fluid bolus during RRT initiation.

Table 2 also summarizes the different RRT prescriptions, achieved ultrafiltration (UF) goals, and durations of treatment across studies.

As reported in Table 3, there was wide variability in the incidence of HIRRT reported by studies within and across different RRT modalities. Detailed results are reported according to RRT modality in Additional file 1. For the four of five IHD studies that reported it, the overall occurrence of HIRRT per session ranged from 10.6% to 65.6% [19–22]. For SLED studies (*n* = 2), the overall occurrence of HIRRT per session was 38.7% [23] and 59.5% [24]. For CRRT studies (*n* = 2), the overall occurrence of HIRRT was not reported on a sessional basis. One CRRT study reported that up to 50% of patients required interventions to treat HIRRT early after initiation of therapy [25] and the other study reported no HIRRT [18]. Study interventions shown to be effective at reducing HIRRT in IHD included: sodium and ultrafiltration profiling (in a study of only 10 patients) [26]; implementation of “guidelines” to limit HIRRT (see Additional file 1 for complete details) [22]; and online blood volume and temperature control by a small observational study [19]. However, online blood volume and temperature control was not found to be effective for IHD patients by a subsequent, larger RCT by the same group [20]. One SLED RCT (*n* = 39 patients) found that lower dialysate temperature in addition to sodium and UF profiling led to less HIRRT [23]. Another SLED RCT did not find that extending SLED duration from 6 to 10 h led to less HIRRT [24]. A crossover RCT (*n* = 30 patients) found that lower temperature at the initiation of CRRT led to improved hemodynamic stability [25], whereas a prospective cohort study (*n* = 21 patients) found no effect in slowing CRRT pump speed at the initiation of treatment [18].

## Discussion

Our systematic review suggests that there is limited evidence with respect to any particular intervention’s efficacy (or lack thereof) in mitigating HIRRT in critically ill patients across RRT modalities. Nonetheless, small studies indicate that the combination of higher dialysate sodium, variable UF rate, and lower temperature might reduce the incidence of HIRRT in critically ill patients with AKI.

Sodium modeling [27] may mitigate intradialytic hypotension (IDH) (a form of HIRRT) in chronic IHD. This is a strategy whereby a dialysis session begins with a high sodium dialysate concentration, which is then reduced in a step-wise manner. Improved hemodynamic tolerance with higher dialysate sodium is believed to be mediated by reducing osmotic fluid shifts between intravascular and interstitial compartments [28]. This can be combined with UF profiling where the UF rate is highest with higher dialysate sodium to maximize fluid removal and is reduced along with dialysate sodium concentration. The study by Lynch et al. [21], using sodium modeling in IHD, was unable to show a significant reduction in HIRRT. Nonetheless, this was a retrospective study where sodium modeling was prescribed by treating clinicians in only 27% of sessions, likely contributing to baseline differences in co-morbidities between the two groups including higher pre-IHD vasopressor requirements in the sodium modeling group. On the other hand, both RCTs that assessed combined sodium and UF profiling did find less HIRRT within the intervention group [23, 26]. In the latter study, which also included cool dialysate in the intervention group, the sample size was small, and the control group had a significantly lower MAP pre- and post-dialysis. As such, based on the available evidence, it remains unclear if sodium profiling alone is a useful technique for limiting HIRRT in the context of AKI and critical illness, but it may be effective in combination with other strategies including UF modeling and cool dialysate.

Possible adverse effects of high dialysate sodium and sodium profiling are reported in the ESRD population on chronic IHD, and include increased thirst, interdialytic weight gain, and hypertension [29–31] which can contribute to left ventricular hypertrophy, cardiovascular events, and increased mortality [29, 32, 33]. A recent systematic review of 23 studies in the chronic IHD population found that higher dialysate sodium led to increased interdialytic weight gains but did not confirm an association with an increased risk of death [34]. The authors concluded that further research is needed to assess the impact of dialysate sodium on mortality [34]. Our included studies reported that post-session sodium levels were similar between groups but did not provide data on adverse effects or fluid balance. This is particularly relevant given the mounting evidence of a strong association between fluid overload and increased mortality in the AKI population [35–38].

There is increasing evidence to support the use of cooled dialysate to limit IDH in outpatient IHD patients [39–42]. In addition, two systematic reviews of cool dialysate in the chronic IHD population did not identify any trials that included an assessment of adverse effects such as mortality, cardiovascular events, access failure, or bleeding [43]. Cooler dialysate promotes vasoconstriction by reducing heat transfer from the dialysate and may mitigate myocardial stunning [39], a phenomenon that has also recently been shown to occur in patients with AKI being treated with IHD [44] and CRRT [45]. The small pilot study by Robert et al. [25] found that decreasing the fluid warmer temperature from 38 °C to 36 °C at the start of CRRT improved hemodynamics but did not impact body temperature. This study did not comment on adverse effects related to hypothermia, but mean body temperature did not fall below 36 °C. Studies involving CRRT have shown that mild decreases in core body temperature result in increased systematic vascular resistance and decreasing oxygen consumption [46, 47]. However, prolonged and extreme hypothermia in the broader ICU population may correlate with organ dysfunction and increased ICU mortality [48]. A study examining the effect of cooling on critically ill febrile patients suggested that hypothermia induced by CRRT results in immune system dysfunction [49] and that an assessment of longer term outcomes in this population is particularly warranted.

Blood volume monitoring has been utilized in the chronic IHD population as a means to predict and thereby prevent hemodynamic instability during treatment [50, 51]. Two prospective observational studies of relative blood volume monitoring in the AKI population on IHD did not find any significant concordance between blood volume monitoring and hypotension [2, 4]. We identified two studies by du Cheyron et al. (2010 and 2013), an observational study [19] followed by an RCT [20], the latter of which found no significant impact on HIRRT or other dialysis-related complications. This suggests that online blood volume monitoring may not have any benefit beyond that which might be provided by cooled dialysate, high dialysate sodium and calcium concentration, and variable UF rate, all of which were part of the standard dialysis prescription. There also may be physiological differences between central and peripheral blood volume, and plasma refilling from dialysis fluid shifts is thought to occur primarily from peripheral rather than central compartments [52]. Consequently, this process may not be reflected in blood volume monitoring from central venous catheters. Interestingly, a recent small study found that low baseline peripheral perfusion index (PPI) measured by pulse oximetry could predict hypotension during continuous venovenous hemofiltration (CVVH) in the ICU [53].

Patients at higher risk of complications, given hemodynamic instability at baseline, are more likely to be selected for treatment with CRRT (or SLED). Hypotension at CRRT initiation has been reported in 18.8–25.0% of patients [8, 54]. Kim et al. [54] also assessed hypotension in relation to CRRT initiation and found that it affected 7.8% of circuit starts. Eastwood et al. [18] compared CRRT routine and slow blood flow rates at initiation and reported no hypotensive events in either arm. However, the study population from Kim et al. as compared to Eastwood et al. was, on average, 10 years older (65.9 ± 11.5 years vs 58 ± 19.9 years) and had a lower MAP at baseline (69.9 ± 9.9 mmHg vs 84.2 ± 15.0 mmHg) (*p* value not provided).

Our systematic review suggests that HIRRT is a common phenomenon across RRT modalities utilized for the treatment of AKI, complicating approximately 10–70% of IHD sessions [19–22, 26], approximately 40–60% of SLED sessions [23, 24], and up to 50% of CRRT sessions [25]. Part of the variability in the frequency of HIRRT observed across studies is most likely attributable to variations in the definition of HIRRT being used, as well as other aspects of how and when different RRT modalities are applied. In comparison, the outpatient IHD definition for IDH has three components: 1) a drop in SBP of 20 mmHg or drop in MAP of at least 10 mmHg; 2) presence of symptoms of end organ ischemia; and 3) intervention carried out by dialysis staff [55]. However, this definition of IDH cannot be readily applied to ICU patients as many are receiving concurrent vasopressor and/or inotropic support, and it is often not possible to assess for ischemic symptoms. This highlights the importance of better defining HIRRT in the context of critical illness as a focus for future research.

The data presented in this systematic review must be interpreted in the context of its limitations. There was substantial heterogeneity among the included studies because of multiple RRT-related interventions, different RRT modalities, and variability in the definition of HIRRT as discussed. Another important limitation is that included studies, and hence our review, did not assess the potential for adverse effects of interventions to limit HIRRT. Also, the timing of the onset of HIRRT within a session was not provided, with the exception of Schortgen et al. [22]*,* and Eastwood et al. [18]. Whether HIRRT occurs at RRT initiation or later during the session has physiological relevance, as one would not expect fluid removal to be the main culprit at session onset. However, rapid fluid shifts between compartments, myocardial stunning, or peripheral vasodilation could precipitate HIRRT early on. With regards to study quality, many RCTs had small sample sizes, and the majority were unblinded. Retrospective studies had important baseline differences between cohorts as interventions were likely prescribed for a clinical reason. The total number of patients from all included studies was only 623 and our systematic review is very likely to have been underpowered to assess most outcomes. This also highlights the extent to which this area is ripe for further study. While this review focused on RRT-related interventions and HIRRT, the impact of different RRT modalities on HIRRT and other outcomes is, unto itself, a controversial aspect of RRT administration in critically ill patients [6]. Nonetheless, the impact of RRT modality on mortality and renal recovery has been the subject of prior reviews [56–60] and was considered beyond the scope of this one.

There are also notable strengths to this study. The search strategy was comprehensive and was conducted according to a previously published protocol [14]. This review indicates that there is a paucity of high-quality evidence to support any particular recommendations for reducing the occurrence of HIRRT in critically ill patients. The most current Kidney Disease Improving Global Outcomes (KDIGO) AKI guidelines do not have recommendations in this regard [56]. French guidelines, based on expert opinion [61], suggest that for critically ill patients the use of higher dialysate sodium concentration, lower dialysate temperature, slow blood flow rates, and bicarbonate buffer be used for IHD. These recommendations accord with the findings of our systematic review. Nonetheless, such interventions (as well as novel ones) warrant more research given that the pathophysiology of HIRRT is particularly complex in critically ill patients [1]. Although a study did show that preload dependence prior to RRT initiation can predict HIRRT [62], there is also evidence that most HIRRT is unrelated to preload dependence [63]. As such, preload reduction from UF may often not be the primary driver of HIRRT in this population [44, 63]. Thus, the role of other potentially modifiable RRT-related factors in provoking HIRRT need to be better defined, with strategies developed and tested to mitigate them.

## Conclusion

We identified only five RCTs and four observational studies that assessed RRT-related interventions aimed at reducing HIRRT among critically ill patients with AKI who received RRT. These studies were generally small, likely underpowered, and mostly of low quality. Overall, there is no definitive evidence to support the routine use of any particular RRT-related intervention to limit HIRRT in this population. However, from the data available, and consistent with some current guidelines [61], the use of higher dialysate sodium or sodium modeling, lower dialysate temperature, and slower blood flow rates for patients at risk of HIRRT should be considered in most cases. The lack of a consistent definition for HIRRT presents an impediment for further study. Establishing a uniform definition of HIRRT that is able to encompass drops in blood pressure as well as interventions taken in response to hemodynamic instability (e.g., fluid boluses, UF cessation) across different RRT modalities will be challenging. Nonetheless, doing so could help facilitate the design and execution of future trials testing interventions to prevent or mitigate HIRRT and its consequences.

## Additional file

Additional file 1: Results According to RRT Modality. (PDF 131 kb)

## Abbreviations

AKI: Acute kidney injury
CRRT: Continuous renal replacement therapy
CVVH: Continuous venovenous hemofiltration
ESRD: End-stage renal disease
HIRRT: Hemodynamic instability related to renal replacement therapy
ICU: Intensive care unit
IDH: Intradialytic hypotension
IHD: Intermittent hemodialysis
KDIGO: Kidney Disease Improving Global Outcomes
MAP: Mean arterial pressure
NOS: Newcastle-Ottawa Scale
PPI: Peripheral perfusion index
RCT: Randomized controlled trial
RRT: Renal replacement therapy
SBP: Systolic blood pressure
SLED: Sustained low-efficiency dialysis
UF: Ultrafiltration
