# Supplementary material for: Interventions to prevent hemodynamic instability during renal replacement therapy in critically ill patients: a systematic review
Source: Crit Care. 2018 Feb 22;22:41. doi: 10.1186/s13054-018-1965-5 (PMC5822560; doi:10.1186/s13054-018-1965-5)
Supplement: Supplementary file 1 — Results According to RRT Modality. (PDF 131 kb) [file 13054_2018_1965_MOESM1_ESM.pdf]

Additional File 1: Results According to RRT Modality

Intermittent Hemodialysis

Five studies that were included for analysis involved intermittent hemodialysis (IHD). Study design, population characteristics, hemodynamic instability during renal replacement therapy (HIRRT) definitions, and renal replacement therapy (RRT) information are summarized in Tables 1, 2 and 3 (main paper). Outcomes according to intervention are summarized in Table S1. Of the five studies involving IHD, two are randomized controlled trials (RCTs), and three are observational studies. They all employed different strategies aimed at reducing HIRRT.

Table S1: IHD study outcomes, grouped according to intervention

<table>
<tr><th>1st Author, Year</th><th>HIRRT Definition</th><th>Outcome</th><th>Control</th><th colspan="2">Case</th><th>p-value</th></tr>
<tr><td colspan="7">Dialysate sodium modeling vs fixed dialysate sodium</td></tr>
<tr><td rowspan="3">Lynch (2015)</td><td rowspan="3">SBP < 80mmHg, or 50mmHg drop from pre-HD BP, and/or start of vasopressor during IHD</td><td>Composite*</td><td>104/161 (64.6%)</td><td colspan="2">16/30 (53.3%)</td><td>NS</td></tr>
<tr><td>% sessions affected by HIRRT</td><td>59/650 (9.1%)</td><td colspan="2">36/242 (14.9%)</td><td>NS</td></tr>
<tr><td>Failed UF goal</td><td>228/650 (35.1%)</td><td colspan="2">118/242 (48.8%)</td><td>NS</td></tr>
<tr><td colspan="7">Variable dialysate sodium and ultrafiltration modeling</td></tr>
<tr><td rowspan="2">Paganini (1996)</td><td rowspan="2">Intervention: volume +/- vasopressors</td><td>Intervention (albumin or pressor)</td><td>45.4%</td><td colspan="2">16%</td><td>P<0.001</td></tr>
<tr><td>Blood volume change</td><td>-7.6</td><td colspan="2">-6.6</td><td>P<0.05</td></tr>
<tr><td colspan="7">Blood volume and temperature control</td></tr>
<tr><td rowspan="4">du Cheyron (2010)</td><td rowspan="4">SBP<90mmHg or fall>40mmHg</td><td>Incidence of HIRRT</td><td>110/383 (28.7%)</td><td colspan="2">41/189 (21.7%)</td><td>NS</td></tr>
<tr><td>Arrhythmias</td><td>22/383 (5.7%)</td><td colspan="2">8/189 (4.2%)</td><td>NS</td></tr>
<tr><td>IV fluids, pressors</td><td>46/189 (24.3%)</td><td colspan="2">103/383 (26.9%)</td><td>NS</td></tr>
<tr><td>Incidence of HIRRT¶</td><td>22/132 (16.6%)</td><td colspan="2">38/132 (28.8%)</td><td>0.005</td></tr>
<tr><td rowspan="2">du Cheyron (2013)</td><td rowspan="2">SBP<90mmHg justifying intervention</td><td rowspan="2">Rate of HIRRT (%)</td><td rowspan="2">17.0</td><td>BVM</td><td>BVM + BTM</td><td rowspan="2">NS</td></tr>
<tr><td>17.3</td><td>14. 9</td></tr>
<tr><td colspan="7">Institution recommendations for HIRRT in AKI in ICU</td></tr>
<tr><td rowspan="4">Schortgen (2000)</td><td rowspan="4">SBP drop>10% from baseline.</td><td>Frequency of HIRRT per session (%)</td><td>27.0</td><td colspan="2">19.0</td><td>0.001</td></tr>
<tr><td>Composite HIRRT, intervention (%)</td><td>71.0</td><td colspan="2">61.0</td><td>0.015</td></tr>
<tr><td>Length of stay (days)</td><td>11.0</td><td colspan="2">7.0</td><td>0.04</td></tr>
<tr><td>Mortality (%)</td><td>53.3</td><td colspan="2">47.3</td><td>NS</td></tr>
</table>

The term 'case' is used to refer to the group that underwent an intervention to limit HIRRT, irrespective of study design.
*Composite of in-hospital death or dialysis dependence at discharge
¶Matched case-controls 1:1 for age, SAPSII, dialysate sodium concentration, ultrafiltration

BVM, blood volume online monitoring; BTM, blood temperature online monitoring

Additional File 1: Results According to RRT Modality

Paganini *et al.* (1996) conducted a randomized cross-over study of 10 IHD patients focused on two interventions: dialysate sodium and ultrafiltration (UF) modeling in combination. Briefly, this technique involves starting the dialysis session with a higher dialysate sodium concentration (160mmol/L in this study), and incrementally reducing it to 140 mmol/L. In this variable group, the UF rate was also decreased over the course of a treatment. Pre- and post-IHD sodium concentrations in the fixed and variable group were 136.6+/-6.0; 139.1+/-3.7mmol/L and 138.7+/-5.1;141.7+/-2.3mmol/L, respectively. Thus, post-session serum sodium levels were similar between groups. This study found a significant reduction in the use of colloid or vasopressor infusions from 45.4% to 16.0% (p<0.001) [1]. Specific hemodynamic parameters were not provided.

One group conducted two studies (first an observational study and then an RCT) investigating blood volume and temperature controlled dialysis. This strategy is based on automatic biofeedback control for both blood volume and temperature changes. In theory, this should prevent temperature rises and rapid decreases in blood volume that depends on the rate of fluid shift from the interstitial compartment during UF. The first by du Cheyron *et al.* (2010) was a prospective observational study comparing 20 ICU patients with monitoring devices to historical controls [2]. In their initial analysis, there was a trend towards a significant reduction in HIRRT (21.7% vs 28.7%; p=0.09), and greater net UF was achieved in the treatment group (3.0 +/- 0.6L vs 2.1 +/- 0.6L; p<0.0001). UF discontinuation occurred similarly in both groups, but the treatment group had less sessions discontinued (4% vs 10%, p=0.02). They also employed a matched case-control analysis using treatment sessions as the unit of analysis, which showed a statistically significant reduction in HIRRT, from 28.8% to16.6% (p=0.005) of dialysis sessions. This group subsequently conducted a

Additional File 1: Results According to RRT Modality

three-arm RCT (A=standard; B=blood volume monitor; C=blood volume and temperature monitor) with 74 patients which found no significant difference in the rates of HIRRT between groups with blood volume and temperature monitoring alone [3]. Of note, the IHD parameters used for all patients in this study included a low temperature dialysate of 36.0ºC, a high dialysate sodium concentration (145mmol/L) and a high dialysate calcium bath of 1.75mmol/L. The blood flow speed was initially set at 200-250 mL/min, and adjusted on an unspecified basis.

The retrospective observational study by Lynch *et al.* (2015) assessed the effect of dialysate sodium modeling alone [4]. Sodium modeling did not reduce the incidence of HIRRT, and had no impact on the amount of UF achieved. Of note, both groups were dialyzed with a 1.25mmol/L calcium bath, but the sodium modeling group was more often dialyzed using cool dialysate (12.0% vs 2.3%, $p<0.001$).

Schortgen *et al.* (2000) conducted a before-after study evaluating the effectiveness of implementing a set of guidelines to reduce HIRRT [5] in the context of IHD. The guidelines were outlined in the study but the frequency of which each recommendation was implemented was not reported. These guidelines included using modified cellulosic membranes, a dialysate sodium concentration of 145 mmol/L or greater, limiting maximum blood flow rate to 150 mL/min, minimizing session time to four hours duration, and setting dialysate temperature at 37˚C or less. For the 'hemodynamically unstable' patients, additional recommendations included cooling dialysate to 35˚C, starting sessions without UF then later adapting the UF rate based on hemodynamics, and discontinuing vasodilator therapy if applicable. At baseline, this study's 'after guideline implementation' cohort was a

Additional File 1: Results According to RRT Modality

sicker population than the 'before guideline implementation' cohort, as per mean SAPSII scores and pre-IHD vasopressor requirements (34% vs 16%, respectively, $p<0.001$). Despite this, the 'after guideline implementation' group had a lower incidence of hemodynamic impairment, defined as a composite of reduction in SBP, intravenous fluid infusion or administration of vasoactive drugs (71% vs 61%, $p=0.015$). The study also assessed drops in systolic blood pressure according to IHD session timing and found that the 'after guideline implementation' group had fewer drops in SBP both at session onset (33% vs 21%, $p=0.002$) and during sessions (68% vs 56%, $p=0.002$). As well, it was found that the observed mortality rate in the 'after guideline implementation' group was less than expected, and their median length of stay was significantly shorter than had been for the 'before guideline implementation' group (11 versus 7 days).

Additional File 1: Results According to RRT Modality

Sustained Low Efficiency Dialysis

We included two studies of different interventions in patients treated with slow low efficiency dialysis (SLED), both of which were RCTs. Table S2 details outcomes according to interventions.

Table S2: Study outcomes with SLED or CRRT grouped according to intervention

1st Author, Year	HIRRT Definition	Outcome	Control	Case	p-value
**SLED Studies**					
**Duration of RRT: Extended daily dialysis for 6hrs (control) vs 10 hrs (intervention)**					
Albino (2014)	SBP <90mmHg MAP<60mmHg	HIRRT (%)	81.5	83.7	NS
		Renal recovery (%)	10.5	16.6	NS
		Death (%)	78.9	77.7	NS
**Dialysate sodium and ultrafiltration (UF) modeling**					
Lima (2012)	SBP<90mmHg MAP<60mmHg Interventions	HIRRT (%)	57.1	23.5	0.009
		Length of stay (d)	16	31	NS
		Mortality (%)	85.7	82.3	NS
**CRRT Studies**					
**Temperature 38ºC (control) vs 36ºC (intervention)**					
Robert (2012)	Fall in MAP>20% or intervention	Change in MAP	+1.2mmHg	+8.9mmHg	0.08
		Change in NE rate	0.0	-0.5	NS
**CRRT start pump speed: routine (control) vs slow (intervention)**					
Eastwood (2012)	Vasopressor use and/or fluid bolus at 10 and 30 min	Change in MAP	No hypotension	No hypotension	NS
		Intervention	No difference	No difference	NS

The term 'case' is used to refer to the group that underwent an intervention to limit HIRRT, irrespective of study design.

SLED, slow low efficiency dialysis; CRRT, continuous renal replacement therapy; SBP, systolic blood pressure; MAP, mean arterial pressure; HIRRT, hemodynamic instability during RRT; NS, not significant; NE, norepinephrine infusion; min, minutes

Albino *et al.* (2014) compared the duration of extended daily dialysis (6 vs 10 hours) [6]. High dialysate sodium concentration (142-148 mEq/L) and low dialysate temperature (35.5ºC) were used for both groups. Overall, HIRRT occurred in 82.6% of patients and in 59.5% of RRT sessions with no significant difference between groups. There was no

Additional File 1: Results According to RRT Modality

difference in mortality (77.7% vs 78.9%, p=0.l86) or renal recovery (16.6% vs 10.5%, p=0.21) between the two groups. However, the extended duration group had significantly more sessions interrupted (9.5% vs 30.1%, p=0.03).

Lima *et al.* (2012) conducted a small single center RCT comparing their standard SLED prescription (which includes high dialysate calcium concentration of 1.75mmol/L) to one with a lower dialysate temperature (35.5˚C vs 37.0˚C) combined with sodium and UF modeling [7]. The serum sodium levels were similar in both groups post-SLED, and the profile group had slightly lower temperature (36.4+/-0.9˚C vs 36.9+/-0.9˚C, p=0.05). They found a significant reduction in the frequency of HIRRT between profiling and control groups: 23.5% and 57.1% of RRT sessions, respectively (p=0.009), however, the control group had a significantly lower MAP pre- and post-dialysis. Session duration was similar between both groups, but the profiling group achieved slightly more ultrafiltration (1.59+/-1.0L vs 2.23+/-1.2L, p=0.04). There was no difference in mortality (82.3% vs 85.7%, p=1.0) or length of stay (31 vs 16 days, p=0.18).

Continuous Renal Replacement Therapy

Two studies tested manoeuvres to prevent HIRRT with continuous renal replacement therapy (CRRT) (RCT: n=1). Studies are outlined in Tables 1, 2 and 3 (main paper) and outcomes according to intervention are provided in Table S2. Robert *et al.* (2012) conducted a prospective crossover randomized study comparing the effect of setting the temperature of a heating device warming blood return from CRRT at 38˚C or 36˚C on hemodynamic parameters during treatment [8]. Body temperature was similar between groups for the duration of the study (36.3+/-0.5˚C and 36.4+/-0.5˚C); however, reducing the

Additional File 1: Results According to RRT Modality

device temperature from 38˚C to 36˚C at the beginning of CRRT in group B was shown to significantly increase mean arterial pressure and decrease vasopressor requirements as compared to maintaining the heating temperature at 38˚C. This hemodynamic effect of lower temperature was not seen in the second phase of the study, where temperature was lowered from 38˚C to 36˚C at six hours after initiation of CRRT.

Eastwood *et al.* (2012) conducted a prospective observational study to assess the hemodynamic impact of routine vs lower blood flow speeds at CRRT initiation (blood flow protocol provided in Table 2). They did not record any episodes of hypotension during the start of treatment in either group[9]. This study was focused on hemodynamics at CRRT initiation, and did not record outcomes beyond thirty minutes into the session.

Additional File 1: Results According to RRT Modality

References

1. Paganini EP, Sandy D, Moreno L, Kozlowski L, Sakai K: **The effect of sodium and ultrafiltration modelling on plasma volume changes and haemodynamic instability in intensive care patients receiving haemodialysis for acute renal failure: a prospective, stratified, randomized, cross-over study**. *Nephrol Dial Transplant* 1996, **11**:32-37.
2. du Cheyron D, Lucidarme O, Terzi N, Charbonneau P: **Blood volume- and blood temperature-controlled hemodialysis in critically ill patients: a 6 month, case-matched, open-label study**. *Blood Purif* 2010, **29**:245-251.
3. du Cheyron D, Terzi N, Seguin A, Valette X, Prevost F, Ramakers M, Daubin C, Charbonneau P, Parienti J: **Use of Online Blood Volume and Blood Temperature Monitoring During Haemodialysis in Critically Ill Patients with Acute Kidney Injury: a Single-Centre Randomized Controlled Trial**. *Nephrol Dial Transplant* 2013, **28**:430-437.
4. Lynch KE, Ghassemi F, Flythe JE, Feng M, Ghassemi M, Celi LA, Brunelli SM: **Sodium Modeling to Reduce Intradialytic Hypotension during Haemodialysis for Acute Kidney Injury in the Intensive Care Unit** *Nephrology* 2015.
5. Schortgen F, Soubrier N, Delclaux C, Thuong M, Girou E, Brun-Buisson C, Lemaire F, Brochard L: **Hemodynamic Tolerance of Intermittent Hemodialysis in Critically Ill Patients: Usefulness of Practice Guidelines**. *Am J Respir Crit Care Med* 2000, **162**:197-202.
6. Albino BB, Balbi AL, Ponce D: **Dialysis complications in acute kidney injury patients treated with prolonged intermittent renal replacement therapy sessions lasting 10 versus 6 hours: results of a randomized clinical trial**. *Artificial Organs* 2015, **39**(5):423-431.
7. Lima EQ, Silva RG, Donadi E, Fernandes AB, J.R. Z, Pinto K, Burdmann EA: **Prevention of Intradialytic Hypotension in Patients with Acute Kidney Injruy Submitted to Sustained Low-Efficiency Dialysis**. *Renal Failure* 2012, **34**(10):1238-1243.
8. Robert R, Méhaud J, Timricht N, Goudet V, Mimoz O, Debaene B: **Benefits of an early cooling phase in continuous renal replacement therapy for ICU patients**. *Annals of Intensive Care* 2012, **2**(40).
9. Eastwood GM, Peck L, Young H, Bailey M, Reade MC, Baldwin I, Bellomo R: **Haemodynamic Impact of a Slower Pump Speed at Start of Continuous Renal Replacement Therapy in Critically Ill Adults with Acute Kidney Injury: A Prospective Before-and-After Study**. *Blood Purif* 2012, **33**:52-58.

Additional File 1: Results According to RRT Modality
